# Protein intake, physical activity and grip strength in European and North American community-dwelling older adults: a pooled analysis of individual participant data from four longitudinal ageing cohorts

**DOI:** 10.1017/S0007114522002033

**Published:** 2023-04-14

**Authors:** Nuno M. P. Mendonça, Linda M. Hengeveld, Nancy Presse, Helena Canhão, Eleanor Simonsick, Stephen B. Kritchevsky, Samaneh Farsijani, Pierrette Gaudreau, Carol Jagger, Marjolein Visser

**Affiliations:** 1 EpiDoC Unit, NOVA Medical School, Universidade Nova de Lisboa, Lisbon, Portugal; 2 Comprehensive Health Research Centre (CHRC), Lisbon, Portugal; 3 Population Health Sciences Institute, Newcastle University, Newcastle-upon-Tyne, UK; 4 Department of Health Sciences, Faculty of Science, Amsterdam Public Health research institute, Vrije Universiteit Amsterdam, Amsterdam, the Netherlands; 5 Health Council of the Netherlands, The Hague, the Netherlands; 6 Research Centre on Aging, CIUSSS de l’Estrie-CHUS, Sherbrooke, QC, Canada; 7 Faculty of Medicine and Health Sciences, University of Sherbrooke, Sherbrooke, QC, Canada; 8 Centre de recherche de l’Institut universitaire de gériatrie de Montréal, Montréal, QC, Canada; 9 National Institute on Aging Intramural Research Program, Baltimore, MD, USA; 10 Sticht Center on Aging, Wake Forest School of Medicine, Winston-Salem, NC, USA; 11 Center for Aging and Population Health, Department of Epidemiology, University of Pittsburgh, Pittsburgh, PA, USA; 12 Department of Medicine, University of Montréal and Research Centre of the University of Montréal Hospital Centre, Montréal, QC, Canada

**Keywords:** Protein, Handgrip strength, Physical activity, Joint models, PROMISS, Older adults, One-stage meta-analysis

## Abstract

Higher dietary protein, alone or in combination with physical activity (PA), may slow the loss of age-related muscle strength in older adults. We investigated the longitudinal relationship between protein intake and grip strength, and the interaction between protein intake and PA, using four longitudinal ageing cohorts. Individual participant data from 5584 older adults (52 % women; median: 75 years, IQR: 71·6, 79·0) followed for up to 8·5 years (mean: 4·9 years, SD: 2·3) from the Health ABC, NuAge, LASA and Newcastle 85+ cohorts were pooled. Baseline protein intake was assessed with food frequency questionnaires and 24-h recalls and categorized into < 0·8, 0·8–<1·0, 1·0–<1·2 and ≥ 1·2 g/kg adjusted body weight (aBW)/d. The prospective association between protein intake, its interaction with PA, and grip strength (sex- and cohort-specific) was determined using joint models (hierarchical linear mixed effects and a link function for Cox proportional hazards models). Grip strength declined on average by 0·018 SD (95 % CI: –0·026, –0·006) every year. No associations were found between protein intake, measured at baseline, and grip strength, measured prospectively, or rate of decline of grip strength in models adjusted for sociodemographic, anthropometric, lifestyle and health variables (e.g., protein intake ≥ 1·2 *v·* < 0·8 g/kg aBW/d: *β* = –0·003, 95 % CI: –0·014, 0·005 SD per year). There also was no evidence of an interaction between protein intake and PA. We failed to find evidence in this study to support the hypothesis that higher protein intake, alone or in combination with higher PA, slowed the rate of grip strength decline in older adults.

Older adults gradually lose muscle mass, muscle strength and physical function with age which increases the risk of falls, frailty, disability and all-cause mortality^(1–3)^. Dietary protein, in excess of the current recommended dietary allowance, 0·8 g/kg body weight (BW)/d according to the European Food Safety Authority^(4)^ and the Institute of Medicine^(5)^, has been proposed to slow the decline of age-related muscle strength in older adults^(6)^. However, the relation between protein intake and grip strength, often used as measure of overall muscle strength, is inconsistent across the literature, and very limited for strength decline over time. Prospective observational studies showed that higher protein intake was associated with higher grip strength^(7–10)^, while some have not^(11,12)^. Higher protein intake has also been associated with a slower rate of decline in grip strength over time^(7,8,10)^, while others have not found similar results^(9,11,12)^.

Several expert groups have proposed that the beneficial effect of higher protein intake on muscle strength may work in synergy with physical activity (PA)^(6,13)^. Since both protein intake and PA stimulate muscle protein synthesis, combining both approaches may better protect muscle mass and muscle strength than each alone^(6,13)^. However, few observational studies have addressed this issue and evidence is inconclusive^(9,14)^. In many instances, lack of statistical power in individual studies does not allow for testing the interaction between protein intake and PA, or for achieving robust estimates^(15)^. We overcome this limitation by pooling individual participant data from multiple ageing cohorts. In this study, we hypothesise that higher protein intake slows down the rate of muscle strength decline in older adults in a dose-dependent manner and has a synergistic effect with PA. In order to test our hypothesis, we investigated the prospective relationship between protein intake, and its interaction with PA, and (decline in) grip strength in four longitudinal cohorts in the PROMISS consortium.

## Methods

### Included cohorts and study population

As part of the PROMISS consortium, four longitudinal prospective observational studies of community-dwelling older adults aged > 55 years were included: (a) The Health, Aging and Body Composition Study (Health ABC) from the USA, (b) The Quebec Longitudinal Study on Nutrition and Successful Aging (NuAge) from Canada, now the NuAge Database and Biobank (database received on May 2019), (c) The Longitudinal Aging Study Amsterdam (LASA) from the Netherlands and (d) The Newcastle 85+ Study from the UK. These studies are described in detail elsewhere^(16–19)^. Briefly, Health ABC is a longitudinal cohort study that included 3075 well-functioning community-dwelling Black and White males and females aged 70–79 years at baseline living in the USA. Participants were recruited from Medicare-eligible residents in the metropolitan areas of Memphis, Tennessee, and Pittsburgh, Pennsylvania, between April 1997 and June 1998 and followed annually (clinic visit) or every 6 months (telephone interview) for 16 years^(16)^. NuAge is a longitudinal cohort that recruited 1793 generally healthy community-dwelling males and females aged 67–84 years living in Montreal and Sherbrooke areas (Quebec, Canada) in 2003–2005 and followed them annually (clinic visit) or every 6 months (telephone interview) for 3 years^(19)^. LASA is an ongoing nationally representative longitudinal study of older males and females aged ≥ 55 years residing in the Netherlands. The study started in 1992/93 (*n* 3107), and participants were followed every 3 years until 2018/2019 (most recent wave; wave J). Two additional cohorts were recruited from the same sampling frames at 10 (2002/2003, *n* 1002) and 20 years (2012/2013, *n* 1023) after the baseline^(17)^. The Newcastle 85+ Study is a longitudinal population-based study that approached all people turning 85 years in 2006/2007 (born in 1921) in Newcastle and North Tyneside, UK. At baseline, there were 845 very old males and females who agreed to a health assessment and a review of their GP records^(18)^, who were re-examined after 18, 36 and 60 months. We used year 2 (baseline), 4, 6, 8 and 10 from Health ABC; T1 (baseline), T2, T3 and T4 from NuAge; wave 3B, the Nutrition and Food-related Behavior sub study (baseline) and wave I from LASA; and phase 1 (baseline), 2, 3 and 4 from the Newcastle 85+ (online Supplementary Fig. 1).

We excluded participants who were institutionalised (*n* 44), had very poor cognitive status (score < 18 in the Mini-Mental State Examination or with diagnosed dementia) and no proxy for dietary assessment (*n* 18), had missing dietary intake data (*n* 800), had very high reported energy intake, that is, > 3500 kcal/d for women or > 4000 kcal/d for men (*n* 52), had no data on BMI (*n* 58) or missing grip strength (*n* 141). The analytic sample at baseline comprised 5584 community-dwelling participants (online Supplementary Fig. 1).

### Dietary assessment

For all studies, data on dietary intake were available at baseline (referred to as wave 1 in our study). Dietary intake was assessed in Health ABC by a 108-item interviewer-administered FFQ reflecting the preceding 12 months in year 2^(20)^, in NuAge by three 24-h recalls (one face to face and two by telephone) on two weekdays and one weekend day in T1^(19)^, in LASA by a self-administered 238-item FFQ reflecting the preceding 4 weeks and collected from fall to spring in the ‘Nutrition and Food-related Behavior Study 2014–2015’ sub study^(21,22)^, and in Newcastle 85+ by two 24-h recalls on two non-consecutive weekdays at least 1 week apart in phase 1^(23,24)^. In all studies, energy and protein intake were calculated by using country-specific food composition databases. In Newcastle 85+ and NuAge, individual intakes of protein and energy were averaged within the two or three recall days, respectively.

Energy intake was transformed into cohort-specific z-scores. For participants with a BMI outside the desirable range for older adults of 22–27 kg/m^2^, BW was adjusted to be within the desired BMI range and calculated as previously described^(25,26)^. By calculating adjusted BW (aBW), we attempted to control for the deficit and excess protein intake needs in underweight and overweight people, respectively. Protein intake was expressed per kg of aBW/d (measured at baseline as well), categorised into < 0·8, 0·8–< 1·0, 1·0–< 1·2 and ≥ 1·2 g/kg BW/d and g/kg aBW/d, and used as exposure. These cut-offs were based on expert recommendations for optimal protein intake^(6,13)^, or currently used recommended dietary allowances for protein (e.g. 0·8 is recommended by the European Food Safety Authority^(4)^ and the Institute of Medicine^(5)^, 1·0 by the European DACH (Germany, Austria and Switzerland) countries^(27)^ and 1·2 by the European Nordic countries^(28)^), or on previously published studies on protein intake in older adults^(9,16,26,29–33)^. All variables are described in Supplementary Table 1.

### Physical activity

In Health ABC, PA was measured by a specifically designed questionnaire as described previously^(34)^. Participants indicated whether they had performed exercise in the past 7 d and for how long they spent in each activity. A metabolic equivalent (MET) value in kcal per week per kilogram of BW was determined for each activity and total PA calculated as MET values for each activity × BW. In NuAge, PA was measured using the validated self-reported Physical Activity Scale for the Elderly (PASE) which asked about the frequency, duration and intensity of activities during the past 7 d^(35)^. The total PA score was calculated as time spent on each activity (in hours per week) × item weights and then summed^(36)^. PA in LASA was measured with a validated questionnaire that estimates the frequency and duration and intensity of specific activities in the previous 14 d^(37)^. MET scores were assigned to each activity based on published MET scores lists^(38)^. The frequency × duration × MET was calculated for each activity, summed over and then divided by 14 d. In Newcastle 85+, a validated purposely designed PA questionnaire included questions on how frequently participants engaged in mildly, moderately and highly energetic activities. The resulting total PA score was calculated as 3 × highly energetic activities + 2 × moderately energetic activities+mildly energetic activities^(39)^. PA at baseline was transformed into cohort-specific tertiles (categorised as low, medium and high) and used to categorise PA for subsequent waves. Lower, medium and higher PA in Health ABC was considered as 0–3·27, 3·28–14·20 and ≥ 14·20 kcal/BW/week, respectively; in NuAge as a PASE score of 0–71·3, 71·4–115·4 and ≥ 115·5, respectively; in Newcastle 85+ as a specialised questionnaire score of 0–2, 3–6 and ≥ 7, respectively; and in LASA as 0–32, 32·1–59 and ≥ 59 MET h/week, respectively.

### Muscle strength

Grip strength was used as an objective measure of upper-body and general muscle strength and was used as our outcome^(40)^. In Health ABC, grip strength was measured twice on each hand with an isometric dynamometer (JAMAR). Participants who underwent recent hand surgery or had severe hand pain were excluded. In NuAge, grip strength was measured three times on each hand with a pneumatic dynamometer (Martin Vigorimeter) and expressed in KPa^(12)^. In LASA, grip strength was measured twice on each hand with a hydraulic dynamometer (Model JAMAR 5030J1). The dynamometer was adjusted for hand size. In Newcastle 85+, grip strength was measured twice (alternating sides) on each hand with an isometric dynamometer (Model A5401, Takei Scientific Instruments)^(9)^. In NuAge and LASA, participants were in a sitting position with arms alongside the body with elbows at 90°, whereas in Health ABC participants were sitting but with arms rested on the table and elbows at 90°. In Newcastle 85+, participants were standing with arms alongside the body and elbows at 90°. In all cohorts, the mean grip strength value of the maximum measurement of each hand was used for analysis. Grip strength measured by dynamometer and measured by vigorimeter shows a high correlation^(41)^. For descriptive purposes, we converted grip strength measured by vigorimeter (in KPa) into kg using a factor of 0·46 as has been previously used^(41)^. Furthermore, since methods to assess grip strength were different between cohorts, grip strength at baseline was transformed into sex- and cohort-specific z-scores, and the mean and sd were used to create z-scores for the other waves (z = (x–µ)/σ) where x stands for raw score, µ for population mean and σ for sd.

### Mortality

Ascertainment of vital status differed between cohorts and ranged from a review of hospital records and obituaries to linkage with the National Mortality Registry. In Health ABC, survival time was calculated as the time between age at year 2 (1998–1999) and age of death (censored at 30 September 2014); in NuAge, survival time was calculated as the time from age at T1 (2003–2005) to age of death (censored at 3 May 2010); in LASA, survival time was calculated as the time from age at wave 3B (2012–2013) to death (censored at 22 July 2018); and in Newcastle 85+, survival time was calculated as the time from age at phase 1 (2006–2007) to age of death (censored at 16 January 2018).

Other sociodemographic, anthropometric, lifestyle and health variables used are described in Supplementary Table 1.

### Statistical analyses

Data cleaning, quality control and harmonisation were performed separately for each cohort prior to merging. In order to be harmonised, grip strength was transformed into sex- and cohort-specific z-scores, energy intake into cohort-specific z-scores, and PA and cognition into cohort-specific tertiles. Harmonisation of other sociodemographic, anthropometric, lifestyle and health variables is described in Supplementary Table 1. All harmonised variables were merged to create one dataset. Normality was assessed by Q–Q plots: normally and non-normally distributed variables are presented as means and standard deviations, and medians and interquartile ranges, respectively, and categorical data as percentages and frequency. To determine the association between protein intake and grip strength, we fitted a hierarchical linear mixed effects models with the *lme4* package (version 1.1-20)^(42)^ and Cox proportional hazards for time-to-event data (mortality or censoring) with the *survival* package (version 2.43-3)^(43)^. Briefly, exposure, outcome, confounders and effect modifiers were selected based on their theoretical and clinical relevance, group imbalance (protein intake categories) and their position in directed acyclic graphs. Models with these variables were then fitted, removed and refitted until the best possible and parsimonious model converged. Clustering by cohort was accounted for by the inclusion of a random term for study membership. If data are missing at random, it is accounted for in linear mixed models, but in a longitudinal cohort with a mean age of 75 years at baseline, attrition is high^(44)^ and failure to account for mortality (data not missing at random) would likely result in biased estimates^(45)^. These outcomes (grip strength and mortality) are typically analysed separately, but joint models analyse the two outcomes together with shared parameters in a single likelihood function (maximum likelihood estimation). We therefore fitted joint models with the *JoineRmeta* package (version 0.1.2) in R v3.6.3^(46)^.

Separate models for the association between protein intake and grip strength were fitted. Model l included id (random effect), time since baseline (random effect) and study membership (time-independent and random effect), protein intake (time-independent) and its interaction with time, age (time-dependent), sex (time-independent), height (time-independent) and education (time-independent); model 2 was adjusted for the previous variables plus smoking (time-independent), energy (time-independent) and alcohol intake (time-independent); model 3 was further adjusted for multimorbidity (time-dependent) and cognitive status (time-dependent); and model 4 was further adjusted for PA (time-dependent; except if the model was stratified by PA level). Models 1–3 were also stratified by PA level. Apart from id, study membership and time since baseline, all terms in the models are fixed effects.

### Sensitivity analyses

As a sensitivity analysis, model 4 (fully adjusted) was re-run with 0·8–< 1·0 g/kg aBW/d of protein intake as referent, or with protein in g/kg BW/d (hence with non-adjusted BW), or with percentage of total energy from protein (%), or with protein intake per MJ of energy, or further adjusted for BMI (time-dependent), or weight (time-dependent), or inputting the missing values for multimorbidity and cognitive status (if this was available at an adjacent observation of the same participant), excluding each of the cohorts from the analysis, or using cohort-specific z-scores of grip strength (not sex-specific). Point estimates and CI were used to assess statistical and clinical significance. Results are presented as *β*s and 95 % CI (determined by refitting the models to 150 bootstrap samples).

## Results

More women and more older adults with a lower cognitive status had missing data on grip strength. All other health and sociodemographic characteristics were similar between those with and those without grip strength (online Supplementary Table 2).

### Protein intake, health and sociodemographic characteristics

The analytic sample consisted of 5584 men (47·4 %) and women (52·6 %) with a median age of 75·0 (interquartile range: 71·6–79·0) years at baseline. Maximum follow-up time was 8·5 years (mean: 4·9, sd: 2·3 years), and maximum survival time until event or censoring was 16·2 years (mean: 7·6, sd: 5·1). Most participants were from the Health ABC study (45·6 %), followed by NuAge (30·8 %), Newcastle 85+ (12·8 %) and LASA (10·8 %) (Table 1 and online Supplementary Fig. 1). At baseline, 27 (*n* 1530), 23 (*n* 1304), 21 (*n* 1195) and 28 % (*n* 1555) of the participants had a protein intake < 0·8, 0·8–< 1·0, 1·0–< 1·2 and ≥ 1·2 g/kg aBW/d, respectively. Most of the participants with protein intake < 0·8 g/kg aBW/d were from the Health ABC study (65·2 %), while most participants with protein intake ≥ 1·2 g/kg aBW/d were from NuAge (37·6 %). Participants with higher protein were more often alcohol drinkers, had higher energy intake and had generally higher PA. For example, 29·3 % of those with protein intake < 0·8 g/kg aBW/d and 39·1 % of those with protein intake ≥ 1·2 g/kg aBW/d had a high level of PA. Age, body height and cognitive status were statistically different between protein intake categories but not clinically significant (Table 1). There was no evidence of a difference in mortality by protein intake category (online Supplementary Fig. 2). Health and sociodemographic characteristics by wave of follow-up, cohort and protein intake categories, and protein intake categories and PA are shown in Supplementary Tables 3–5. Grip strength was lowest in Newcastle 85+ (17·9 (13·4, 23·9) kg) and highest in LASA (31·5 (24·5, 43·5) kg) at baseline (online Supplementary Table 3). Grip strength decreased from 28·0 (21·5, 35·8) at baseline to 25·0 (20·0, 32·9) kg at wave 5 (last follow-up time point), which meant a decrease of 0·45 (0·94) sds over the follow-up (online Supplementary Table 4).

**Table 1.** Health and sociodemographic characteristics of participants by protein intake category (g/kg aBW/d) at baseline and muscle strength during follow-up (Numbers and percentages)

	All (*n* 5584)		< 0·8 (*n* 1530)		0·8–< 1·0 (*n* 1304)		1·0–< 1·2 (*n* 1195)		≥ 1·2 (*n* 1555)		
	%	*n*	%	*n*	%	*n*	%	*n*	%	*n*	*P*
Sociodemographic											
Age (years)											
Median	75·0		75·0		75·0		75·0		74·0		< 0·001
IQR	71·6, 79·0		72·0, 79·0		72·0, 79·5		71·0, 79·0		70·8, 79·0		
Women	52·6	2939	53·5	819	53·2	694	53·3	637	50·7	789	0·372
Cohort											< 0·001
Health ABC	45·6	2547	65·2	997	44·2	576	37·2	444	34·1	530	
NuAge	30·8	1720	16·7	256	33·1	431	37·6	449	37·6	584	
LASA	10·8	601	5·0	77	7·9	103	13·4	160	16·8	261	
N85+	12·8	716	13·1	200	14·9	194	11·9	142	11·6	180	
Education											0·074
Lower	31·8	1776	31·1	474	33·4	435	29·9	357	32·8	510	
Medium	37·8	2107	37·0	564	36·3	474	38·1	454	39·5	615	
Higher	30·4	1695	32·0	488	30·3	395	32·0	382	27·7	430	
Anthropometry											
Height (m)											
Mean	1·65		1·66		1·65		1·65		1·64		< 0·001
sd	0·10		0·10		0·09		0·10		0·10		
BMI (kg/m^2^)											
Mean	27·0		27·7		27·1		26·9		26·3		< 0·001
sd	4·8		4·5		4·7		4·7		5·0		
Lifestyle											
Smokers	8·6	480	8·1	123	8·4	110	8·7	104	9·2	143	0·712
Alcohol drinkers	44·6	2492	38·0	581	42·8	558	48·5	579	49·8	774	< 0·001
Physical activity											< 0·001
Lower	32·2	1797	36·5	558	30·2	393	32·6	389	29·4	457	
Medium	33·7	1882	34·2	523	35·2	458	34·4	411	31·5	490	
Higher	34·1	1901	29·3	449	34·6	451	33·0	394	39·1	607	
Health											
Multimorbidity	50·8	2702	49·4	736	52·2	642	52·9	593	49·4	731	0·150
Cognition											0·022
Lower	29·6	1607	31·0	453	29·7	378	30·0	351	28·0	425	
Medium	41·8	2267	39·4	575	40·9	520	40·7	476	45·8	696	
Higher	28·6	1548	29·6	433	29·3	373	29·3	343	26·2	399	
Dietary intake											
Energy intake, z-score											
Mean	0·00		–0·81		–0·18		0·16		0·84		< 0·001
sd	1·00		0·65		0·72		0·76		0·94		
Protein (g/d)											
Mean	70·0		44·1		62·3		75·2		97·9		< 0·001
sd	24·7		10·9		9·1		10·7		20·4		
Protein (% energy)	15·3	3·3	13·6	2·8	14·8	3·0	15·8	2·9	17·1	3·2	< 0·001
Protein (g/kg aBW/d)											
Mean	1·0		0·6		0·9		1·1		1·5		< 0·001
sd	0·4		0·1		0·1		0·1		0·3		
Muscle strength											
Grip strength (kg)	Median	IQR	Median	IQR	Median	IQR	Median	IQR	Median	IQR	
Baseline	28·0	21·5, 35·8	27·6	21·0, 36·0	27·1	21·0, 35·0	28·0	21·7, 35·0	28·5	22·5, 36·3	0·001
Wave 2	27·1	21·0, 35·0	27·0	21·0, 35·0	27·0	21·0, 34·5	27·6	21·0, 35·0	27·6	21·9, 35·0	0·140
Wave 3	26·0	20·0, 33·4	26·0	20·0, 34·0	26·2	20·0, 33·0	26·9	20·7, 34·0	26·0	20·0, 33·0	0·431
Wave 4	25·3	19·8, 32·2	25·0	19·8, 33·1	25·0	19·0, 31·3	26·0	20·6, 33·0	25·8	19·3, 32·2	0·110
Wave 5	25·0	20·0, 32·0	25·0	20·0, 32·0	24·0	19·0, 31·0	25·0	20·0, 33·0	25·0	19·0, 31·0	0·277
Grip strength, z-score	Mean	sd	Mean	sd	Mean	sd	Mean	sd	Mean	sd	
Baseline	0·00	1·00	0·02	0·99	–0·04	1·01	–0·02	0·99	0·03	1·01	0·157
Wave 2	–0·10	0·99	–0·07	0·98	–0·11	0·99	–0·10	1·01	–0·11	1·00	0·604
Wave 3	–0·21	1·01	–0·20	0·97	–0·21	1·02	–0·20	1·01	–0·22	1·04	0·281
Wave 4	–0·36	1·01	–0·31	0·99	–0·39	0·99	–0·34	1·03	–0·38	1·02	0·074
Wave 5	–0·45	0·94	–0·43	0·96	–0·48	0·90	–0·42	0·99	–0·48	0·94	0·732

aBW, adjusted body weight; IQR, interquartile range; Health ABC, Health, Aging and Body Composition Study; NuAge, Quebec Longitudinal Study on Nutrition and Successful Aging; LASA, Longitudinal Aging Study Amsterdam; N85+, Newcastle 85+ Study.

Cognition was assessed with the Mini-Mental State Examination. Smokers and alcohol drinkers represent current consumers. Z-scores and tertiles are cohort-specific, and z-scores for grip strength are also sex-specific. Protein (% energy) refers to the percentage of total energy from protein.

### Protein intake and muscle strength

**Fig. 1.**
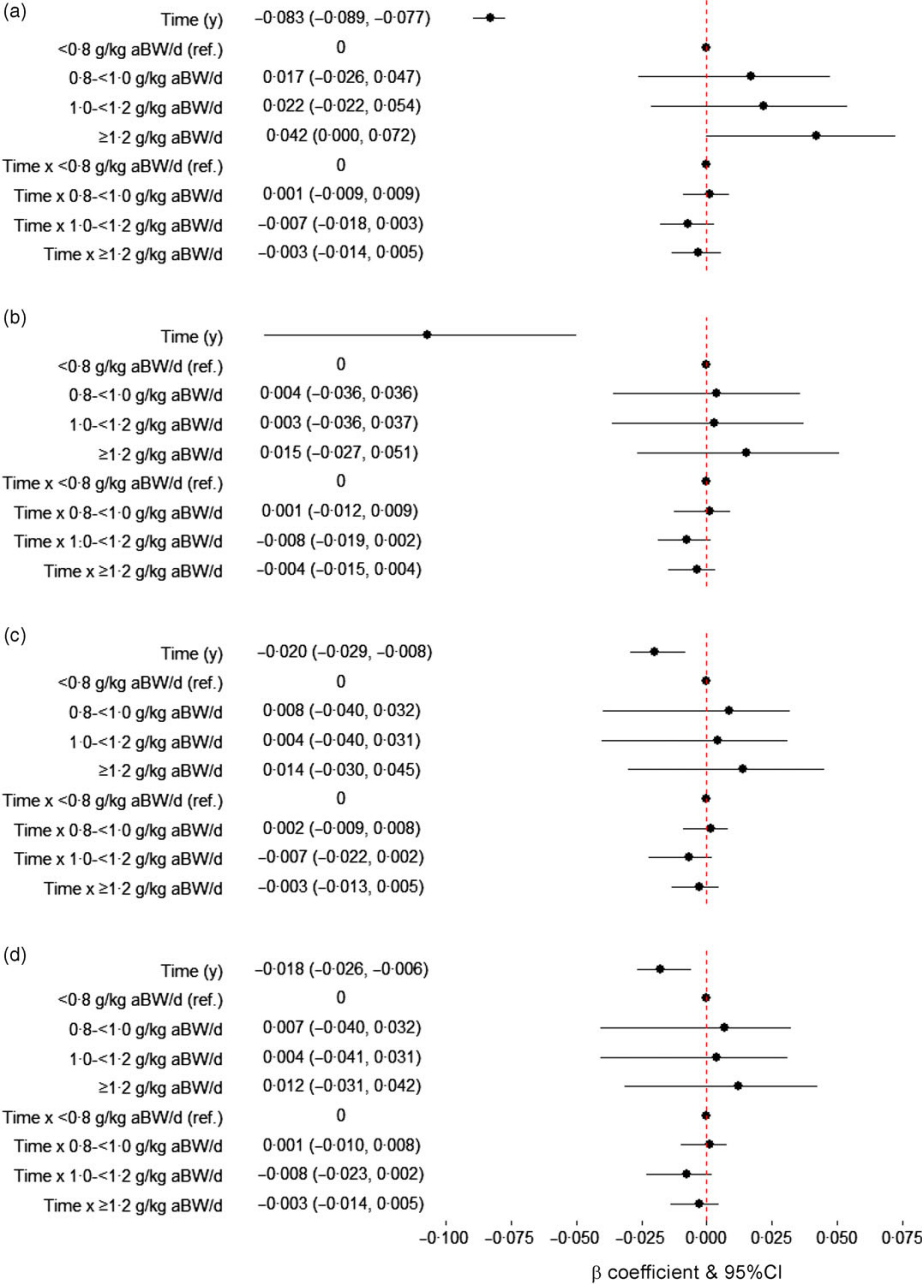
Association between protein intake (g/kg aBW/d) at baseline and grip strength (sex- and cohort-specific z-score) over time. Model 1 (a) is adjusted for sex, age, education and height (*n* 18809 person-years). Model 2 (b) is further adjusted for smoking and, energy and alcohol intake (*n* 18794 person-years). Model 3 (c) is also adjusted for cognition (Mini-Mental State Examination) and multimorbidity (*n* 18663 person-years), and model 4 (d) is further adjusted for physical activity (*n* 18643 person-years). Results are presented as *β* coefficients and 95 % CI in the x-axis and the terms of interest in the y-axis. The *β* coefficient and 95 % CI for the term time (y) in panel (B) is –0·107 (–0·169, –0·050). educ, Education; g/kg aBW/d, grams of protein per kilogram of adjusted body weight per d; ref, referent.

In our models, sex- and cohort-specific grip strength declined on average by 0·018 sd (95 % CI –0·026, –0·006) every year since baseline. We found no associations between protein intake (< 0·8 (referent), 0·8–< 1·0, 1·0–< 1·2 and ≥ 1·2 g/kg aBW/d and g/kg) and grip strength (sex- and cohort-specific z-score) and rate of decline of grip strength after adjustment for sex, age, education and height (model 1). The results were similar in more complex models (models 2–4) further adjusted for smoking, energy and alcohol intake, cognition, multimorbidity and PA (e.g. model 4; protein intake ≥ 1·2 *v.* 0·8 g/kg aBW/d: *β* = –0·003, 95 % CI –0·014, 0·005 sd per year) (Fig. 1).

Final models were re-run with 0·8–< 1·0 g/kg aBW/d of protein intake as referent, or with protein in g/kg BW/d (hence with non-adjusted BW), or with percentage of total energy from protein (%), or with protein intake per MJ of energy (online Supplementary Table 6), or further adjusted for BMI (time-dependent), or weight (time-dependent), or inputting the missing values for multimorbidity and cognitive status (if these were available at an adjacent observation of the same participant), or excluding each of the cohorts from the analysis (online Supplementary Fig. 3), or using cohort-specific z-scores of grip strength (not sex-specific), but none of these substantially changed the results. For example, participants with protein intake < 0·8, 1·0–< 1·2 and ≥ 1·2 g/kg aBW/d had similar rates of grip strength decline than those with 0·8–< 1·0 g/kg aBW/d (*β* = –0·001, 95 % CI –0·010, 0·007; *β* = –0·009, 95 % CI –0·020, 0·002; *β* = –0·004, 95 % CI –0·014, 0·005 sd per year, respectively). However, it is worth noting that, although not significant, the sensitivity analysis excluding HABC from the fully adjusted model resulted in the change of direction of the estimates for protein intake (not rate of decline) (online Supplementary Fig. 3).

### Interaction between protein intake and physical activity

**Fig. 2.**
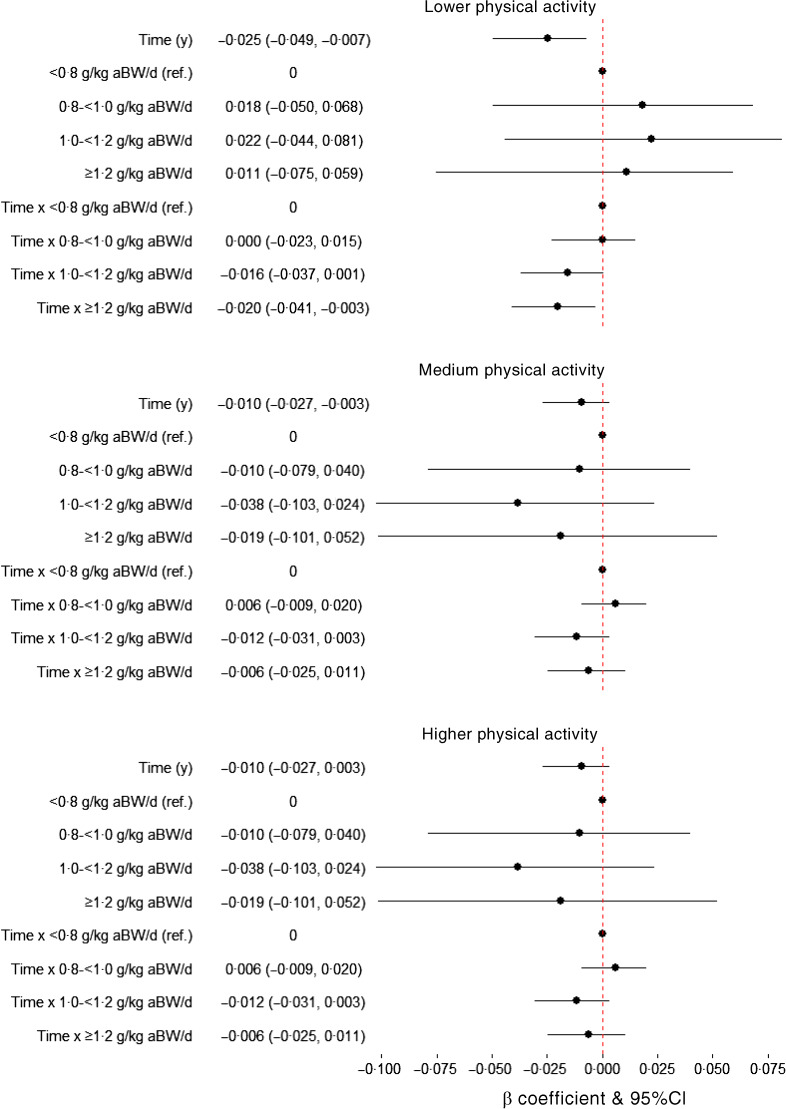
Association between protein intake (g/kg aBW/d) at baseline and grip strength (sex- and cohort-specific z-score) over time by physical activity category. The models are adjusted for sex, age, education, height, smoking, energy and alcohol intake, cognition, multimorbidity and stratified by physical activity (PA) category at baseline (lower PA: *n* 5583 person-years; medium PA: *n* 6411 person-years; higher PA: *n* 6702 person-years). Results are presented as *β* coefficients and 95 % CI in the x-axis and the terms of interest in the y-axis. g/kg aBW/d, grams of protein per kilogram of adjusted body weight per d; ref, referent.

We found no clear interaction between protein intake and PA (all possible interactions *P* > 0·05, e.g. protein intake ≥ 1·2 g/kg aBW/d × high PA, *β*: –0·013, 95 % CI –0·113, 0·059). We also stratified the fully adjusted models by PA (low, medium and high) and found no strong evidence of protein intake being associated with grip strength in any PA category (Fig. 2). However, there was trend for higher protein intake to be associated with faster decline in grip strength within participants with low PA but not within medium or high PA (e.g. time × ≥ 1·2 g/kg aBW/d *v.* time × < 0·8 g/kg aBW/d protein intake; *β* = –0·020, 95 % CI –0·041, –0·003 sd per year).

## Discussion

We found no associations in this study between protein intake measured at baseline (expressed as g/kg aBW/d) and grip strength and rate of decline of grip strength over a maximum follow-up of 8·5 years in community-dwelling older adults. Following from this, we also did not find evidence of an interaction between protein intake and PA in this pooled analysis of individual participant data from four longitudinal ageing cohorts.

In previous analyses of one of the individual cohorts of the pooled analysis, NuAge, higher protein intake was not associated with the 3-year change in grip strength or knee extensor strength^(12)^ nor with rate of decline in grip strength in Newcastle 85+ over 5 years^(9)^. However, higher protein intake was associated cross-sectionally with higher knee extensor strength in NuAge (but not grip strength) at the last data collection^(12)^ and with higher grip strength in Newcastle 85+^(9)^. Further, energy-adjusted protein intake was associated with muscle strength score (sex-specific sum of handgrip, elbow flexors and knee extensor strength) in NuAge as well^(47)^. In Health ABC, no analysis of protein intake and grip strength has been conducted, but higher protein intake was associated with reduced lean mass and appendicular lean mass decline over 3 years^(48,49)^, although, not with the change in mid-thigh muscle cross-sectional area and appendicular lean mass over 5 and 6 years, respectively^(49,50)^. We did not observe any association between protein intake and grip strength in this pooled individual data from the Health ABC, NuAge, LASA and Newcastle 85+. Prospective observational studies (cohorts not included in our study) on protein intake and grip strength in older adults are inconsistent with most finding a protective effect^(7,8,10)^, while others did not^(11)^. There are a few important differences that may explain the different results, namely that Beasley *et al.* ^(8)^ and Mclean *et al.* ^(7)^ only recruited women, that Beasley *et al.* used protein intake adjusted for energy intake with the residual method^(8)^, that Mclean *et al* calibrated the FFQ for doubly labelled water and 24-h urinary nitrogen^(7)^, and that Isanejad *et al* used 3-d food records^(10)^. Our analysis adjusted for energy intake or used protein intake expressed by % of total energy or by 1 MJ of energy, but it is possible that residual confounding remained.

Previously, we showed that participants (pooled analysis of the same cohorts) with protein intake ≥ 0·8 g/kg aBW/d had slower decline in walking speed and were less likely to report incident mobility limitations, and in a dose-dependent manner^(51)^. This discrepancy with our previous findings may be because higher protein intake may be more relevant to physical function than to handgrip strength alone. Using a different measure of muscle strength than grip strength, such as knee extensor strength, or a combination of measures reflecting overall muscle strength might have yielded different results. In fact, the muscles are required to perform a grip strength test, but a small proportion of the overall muscle mass and a significant part of the decline in grip strength with ageing appear to relate to neuromuscular activation rather than contractile volume^(52)^.

In the fully adjusted models, grip strength declined on average by 0·018 sd per year. Original scores are from different distributions (so caution interpreting the back-transformation is needed), but using the mean (28·9 kg) and sd (10·3 kg) at wave 1, grip strength decline would be equivalent to a decline of 0·19 kg/ year or 0·9 kg (3 %) over 4·9 years. This is slightly lower than younger participants (mean: 66·0 years, sd:9·1) from the English Longitudinal Study of Ageing who lost on average 0·03 sd of grip strength every year which equated to a reduction of 1·6 kg (6 %) in women and 2·3 kg (5 %) in men over 9 years^(53)^. Beasley *et al.* found that older women from the Women’s Health Initiative lost, on average, 3·8 % of the baseline grip strength over 7 years^(8)^, and Mclean *et al* found that older adults in the Framingham Offspring Cohort lost, on average, 1·6 % of the baseline grip strength over almost 6 years^(7)^. It is possible that a more pronounced decline in grip strength in our study or longer follow-up would have been necessary to observe an association with protein intake. In fact, the minimal clinically important difference for grip strength is somewhere between 5·0 and 6·5 kg which is considerably higher than the mean grip strength decline in our study^(54)^.

Future analysis of secondary data should consider strata of grip strength decline and/or extending the follow-up time during study design.

Previously in the Newcastle 85+, three dietary pattens were derived: a high red meat, a low meat (under-represented by meat but participants had the highest consumption of fruits, nuts, whole grains and fish) and a high butter dietary pattern. Very old adults with a high red meat dietary pattern had the highest protein intake (non-adjusted) and highest % of energy from protein of the three dietary patterns^(55)^. However, those with the high red meat dietary pattern had worse grip strength (but not worse decline) than those with a low meat dietary pattern^(56)^. Although our analyses adjusted for several confounders, namely energy intake, several other dietary factors may affect muscle strength and the cumulative and synergistic effect of the complex mixture of foods may offer an alternative explanation for the null findings in this study.

We did not find a clear indication for effect modification by PA or a synergistic effect of protein and PA. A 2018 systematic review and meta-analysis of randomised controlled trials also failed to find a synergistic effect of protein supplementation and resistance exercise on muscle strength in non-frail community-dwelling older adults^(14)^. Muscle protein synthesis may be further stimulated if protein intake occurs in closer temporal proximity to exercise and especially if it involves resistant training^(6)^. However, in this pooled analysis, we could not determine when protein intake or PA occurred with an acceptable degree of precision and accurately distinguish between exercise types. Furthermore, although PA was transformed into cohort-specific z-scores prior to analyses, it is possible that this transformation was not enough to deal with all residual differences. For example, PA was estimated in NuAge for the previous 7 d, while in LASA this was asked for the previous 14 d. A major strength of this study is that we harmonised data from four large ageing cohorts and performed an individual participant pooled analysis, which allowed us to significantly increase our sample size and test for interactions that we could not test in individual cohorts through stratification by PA level. For example, there were 103 participants in LASA with a protein intake of 0·8–1·0 g/kg aBW/d at baseline, and of those, 31, 32 and 40 had lower, medium and higher PA, respectively. A model that tested the interaction between protein intake and PA in a single cohort like LASA would have resulted in considerably more unprecise estimates than those we report. The use of an objective measure of muscle strength (grip strength), the large range of covariates adjusted for, and the use of joint modelling to account for non-random attrition and study membership are other major strengths of this study. One important, yet common limitation, is that protein intake was measured at baseline only and assumed to be stable or have declined proportionally over time. If that assumption does not hold, non-differential misclassification of protein intake during follow-up might have occurred and may have biased the results towards the null. Misreporting is a common limitation for self-reported methods, especially underreporting in dietary intake. Although protein-rich foods are not usually underreported^(57)^, it is possible that protein intake was misclassified and biased the association with grip strength towards the null. Additionally, although protein intake was categorised as part of the harmonisation process, dietary intake was assessed by FFQ in Health ABC and LASA, and with multiple 24-h recalls in NuAge and Newcastle 85+. These two methods may give slightly different estimates, resulting is misclassification. We also did not distinguish between animal and vegetable protein which may have yielded a different result than total protein. In fact, Mclean *et al.* found that older adults with higher total or animal protein intake had lower declines in grip strength but failed to see the same for vegetable protein intake^(7)^. Body composition is a major driver of grip strength and as such, protein was expressed per kg of BW, analyses were adjusted for height, and sensitivity analyses further adjusted for weight or BMI. However, measures such as appendicular lean mass or fat-free mass were not available for all cohorts and may have been important effect modifiers.

### Conclusions

We found no convincing evidence in this study that protein intake (measured at baseline and expressed as g/kg aBW/d) was associated with grip strength over time in community-dwelling older adults, or that there was an interaction between protein intake and PA. It is possible that a higher grip strength decline or longer follow-up was needed to observe an association.

## Supplementary material

For supplementary material accompanying this paper visit https://doi.org/10.1017/S0007114522002033. click here to view supplementary material
