# Supplementary material for: Protein intake, physical activity and grip strength in European and North American community-dwelling older adults: a pooled analysis of individual participant data from four longitudinal ageing cohorts
Source: Br J Nutr. 2022 Jul 6;129(7):1221–31. doi: 10.1017/S0007114522002033 (PMC9816353; doi:10.1017/S0007114522002033)
Supplement: Supplementary file 1 [file S0007114522002033sup001.docx]

**Supplementary Material**

***Protein intake, physical activity and grip strength in European and North American community-dwelling older adults: a pooled analysis of individual participant data from four longitudinal ageing cohorts***

Nuno Mendonça ^1-4^, Linda M. Hengeveld ^4,5^, Nancy Presse ^6-8^, Helena Canhão ^1,2^, Eleanor M. Simonsick ^9^, Stephen B. Kritchevsky ^10^, Samaneh Farsijani ^11^, Pierrette Gaudreau ^12^, Carol Jagger ^3^, Marjolein Visser ^4^

^1^ EpiDoC Unit, CEDOC, NOVA Medical School, Universidade Nova de Lisboa, Lisbon, Portugal, ^2^ Comprehensive Health Research Centre (CHRC), NOVA Medical School, Universidade Nova de Lisboa, Portugal, ^3^ Population Health Sciences Institute, Newcastle University, Newcastle-upon-Tyne, UK, ^4^ Department of Health Sciences, Faculty of Science, Amsterdam Public Health research institute, Vrije Universiteit Amsterdam, Amsterdam, the Netherlands, ^5^ Health Council of the Netherlands, The Hague, the Netherlands, ^6^ Research Centre on Aging, CIUSSS de l’Estrie-CHUS, Sherbrooke, Quebec, Canada, ^7^ Faculty of Medicine and Health Sciences, University of Sherbrooke, Sherbrooke, Quebec, Canada,^8^ Centre de recherche de l’Institut universitaire de gériatrie de Montréal, Montréal, Quebec, Canada,^9^ National Institute on Aging Intramural Research Program, Baltimore, Maryland, USA, ^10^ Sticht Center on Aging, Wake Forest School of Medicine, Winston-Salem, North Carolina, USA, ^11^ Center for Aging and Population Health, Department of Epidemiology, University of Pittsburgh, Pennsylvania, USA, ^12^ Department of Medicine, University of Montréal and Research Centre of the University of Montréal Hospital Centre, Montréal, Quebec, Canada.

*****Corresponding author: Nuno Mendonça, address: NOVA Medical School, Universidade Nova de Lisboa, Rua do Instituto Bacteriológico, nº5, 1150-082 Lisboa, Portugal, email: nuno.mendonca@nms.unl.pt, telephone: +351218803110

**Supplementary Table 1.** Description and operationalization of selected variables.

| **Variable** | **Description** | **Wave**^1^ | **Coding** |
| --- | --- | --- | --- |
| id | (Pseudo-)anonymous unique identifier | Baseline | string |
| Study | Cohort identifier | Baseline | 1=Health ABC  2=NuAge  3=LASA  4=Newcastle 85+ |
| **Exposure** | | | |
| Protein intake (non-adjusted) | Protein intake per non-adjusted body weight per day (g/kg BW/d) | Baseline | 0=<0.8 g/kg BW/d  1=0.8-<1.0 g/kg BW/d  2=1.0-<1.2 g/kg BW/d  3=≥1.2 g/kg BW/d |
| Protein intake (adjusted) | Protein intake per adjusted body weight per day (g/kg aBW/d) | Baseline | 0=<0.8 g/kg aBW/d  1=0.8-<1.0 g/kg aBW/d  2=1.0-<1.2 g/kg aBW/d  3=≥1.2 g/kg aBW/d |
| **Outcome** | | | |
| Grip Strength | Sex and cohort-specific z-scores of grip strength measured in kg and KPa | Baseline, wave 2,3,4,5 | numerical |
| Mortality | Death events | NA | 0=alive by censoring date  1=dead |
| Survival age | Age at death or censoring | Baseline, wave 2,3,4,5 | numerical |
| **Possible Confounders** | | | |
| Age | Age in years since date of birth | Baseline, wave 2,3,4,5 | numerical |
| Sex | Self-reported biological sex | Baseline | 0=man  1=woman |
| Education | Self-reported highest level of education attained (HABC, LASA) or total number of years of full-time education (NuAge, N85+) | Baseline | 0=lower or ≤9 years  1= intermediate or 10-14 years  2=higher or ≥15 years |
| Height | Height measured in (m) | Baseline | numerical |
| Body mass index | Measured body weight (kg)/ height (m)^2^ | Baseline, wave 2,3,4,5 | numerical |
| Smokers | Whether participants currently smoke | Baseline | 0=non-smoker  1=smoker |
| Energy intake | Cohort-specific z-scores of total energy intake (MJ) per day | Baseline | numerical |
| Alcohol intake | Whether participants currently drink alcohol | Baseline | 0=non-drinker  1=drinker |
| Cognition | Cohort-specific tertiles of Mini-Mental State Examination (MMSE) (NuAge, LASA and N85+) [1] or an extension with a 100-point scale (Teng 3MS) (HABC) [2] | Baseline, wave 2,3,4,5 | 0=lower MMSE  1=medium MMSE  2=higher MMSE |
| Multimorbidity | Co-occurrence of two or more chronic diseases^2^ | Baseline, wave 2,3,4,5 | 0=< 2 chronic diseases  1=≥ 2 chronic diseases |
| Physical activity | Cohort-specific tertiles of self-reported physical activity | Baseline, wave 2,3,4,5 | 0=lower physical activity  1=medium physical activity  2=higher physical activity |

^1^ Not all waves are at the same time of follow-up among cohorts. In Health ABC the necessary variables were available at year 2 (operationalized as baseline), 4 (wave 2), 6 (wave 3), 8 (wave 4) and 10 (wave 5). In NuAge data were available at year 1 (baseline), 2 (wave 2), 3 (wave 3) and 4 (wave 4). In LASA variables were available at wave 3B (baseline) and at wave I after 3 years (wave 2). Newcastle 85+ has data at baseline, after 18 months (wave 2), after 36 months (wave 3) and after 60 months (wave 4). ^2^ Disease burden in Health ABC was determined by creating a disease score from self-reported cancer (except non-melanoma skin cancer), cardiac diseases (congestive heart disease and myocardial infarction), cerebrovascular diseases (stroke), hypertension (systolic blood pressure ≥130 mmHg and diastolic blood pressure ≥85 mm Hg) and diabetes mellitus (fasting blood glucose ≥126 mg/dl). In NuAge, a disease score was created by summing the presence of self-reported respiratory diseases (asthma, emphysema, and chronic bronchitis), arthritis or rheumatism, hypertension, cardiac diseases, diabetes mellitus, transient ischemic attack, stroke and cancer. In LASA, the seven most common chronic diseases in the Netherlands were assessed by asking about the presence of lung disease, cardiac disease, peripheral arterial disease, diabetes mellitus, stroke, osteoarthritis or rheumatoid arthritis and cancer (except non-melanoma skin cancer) plus hypertension. Lastly, in Newcastle 85+, general practitioner records were reviewed for the presence or absence of cardiac disease, arthritis, hypertension, respiratory disease, cerebrovascular disease, diabetes mellitus and cancer (excluding non-melanoma skin cancer).

**Supplementary Table 2.** Health and sociodemographic characteristics of participants by having or not having data on grip strength at baseline.

|  | **No GS** (*n*=141) | **GS** (*n*=5584) | *p* |
| --- | --- | --- | --- |
| *Sociodemographic* |  |  |  |
| Age, y, median (IQR) | 75.0 (72.0, 77.0) | 75.0 (71.6, 79.0) | 0.138 |
| Women, % (*n*) | 68.1 (96) | 52.6 (2939) | <0.001 |
| Cohort, % (*n*) |  |  | <0.001 |
| Health ABC | 80.1 (113) | 45.6 (2547) |  |
| NuAge | 4.3 (6) | 30.8 (1720) |  |
| LASA | 13.5 (19) | 10.8 (601) |  |
| N85+ | 2.1 (3) | 12.8 (716) |  |
| Education, % (*n*) |  |  | 0.746 |
| Lower | 32.6 (46) | 31.8 (1776) |  |
| Medium | 34.8 (49) | 37.8 (2107) |  |
| Higher | 32.6 (46) | 30.4 (1695) |  |
| *Anthropometry* |  |  |  |
| Height, m, mean (SD) | 1.64 (0.10) | 1.65 (0.10) | 0.082 |
| BMI, kg/m^2^, mean (SD) | 27.5 (4.8) | 27.0 (4.7) | 0.296 |
| *Lifestyle* |  |  |  |
| Smokers, % (n) | 6.0 (8) | 8.6 (480) | 0.368 |
| Alcohol drinkers, % (*n*) | 37.6 (53) | 44.6 (2492) | 0.115 |
| Physical Activity, % (*n*) |  |  | 0.106 |
| Lower | 36.9 (52) | 32.2 (1797) |  |
| Medium | 37.6 (53) | 33.7 (1882) |  |
| Higher | 25.5 (36) | 34.1 (1901) |  |
| *Health* |  |  |  |
| Multimorbidity, % (*n*) | 58.6 (82) | 50.8 (2702) | 0.084 |
| Cognition, % (*n*) |  |  | 0.054 |
| Lower | 39.0 (53) | 29.6 (1607) |  |
| Medium | 38.2 (52) | 41.8 (2267) |  |
| Higher | 22.8 (31) | 28.6 (1548) |  |
| *Dietary intake* |  |  |  |
| Energy intake, z-score, mean (SD) | -0.1 (1.0) | 0.0 (1.0) | 0.287 |
| Protein, g/d, mean (SD) | 66.1 (24.4) | 70.0 (24.7) | 0.064 |
| Protein, g/kg aBW/d, mean (SD) | 1.0 (0.4) | 1.0 (0.4) | 0.282 |

Cognition was assessed with the Mini-Mental State Examination. Smokers and alcohol drinkers represent current consumers z-scores and tertiles are cohort-specific and z-scores for grip strength are also sex-specific. aBW, adjusted body weight; BMI, body mass index; Health ABC, Health, Aging and Body Composition Study; IQR, interquartile range; LASA, Longitudinal Aging Study Amsterdam; MMSE, Mini-Mental State Examination; N85+, Newcastle 85+ Study; NuAge, Quebec Longitudinal Study on Nutrition and Successful Aging; SD, standard deviation; GS, grip strength; y, years.

**Supplementary Table 3.** Health and sociodemographic characteristics of participants by cohort and by protein intake category (g/kg aBW/d) at baseline.

|  | **Health ABC** | | | | | **NuAge** | | | | | **LASA** | | | | | **Newcastle 85+** | | | | |
| --- | --- | --- | --- | --- | --- | --- | --- | --- | --- | --- | --- | --- | --- | --- | --- | --- | --- | --- | --- | --- |
|  | **All** (*n=*2547) | **<0.8** (*n=*997) | **0.8-<1.0** (*n*=576) | **1.0-<1.2** (*n*=444) | **≥1.2** (*n*=530) | **All** (*n*=1720) | **<0.8** (*n=*256) | **0.8-<1.0** (*n*=431) | **1.0-<1.2** (*n*=449) | **≥1.2** (*n*=584) | **All** (*n*=601) | **<0.8** (*n=*77) | **0.8-<1.0** (*n*=103) | **1.0-<1.2** (*n*=160) | **≥1.2** (*n*=261) | **All** (*n*=716) | **<0.8** (*n=*200) | **0.8-<1.0** (*n*=194) | **1.0-<1.2** (*n*=142) | **≥1.2** (*n*=180) |
| *Sociodemographic* | | |  |  |  |  |  |  |  |  |  |  |  |  |  |  |  |  |  |  |
| Age, y, median (IQR) | 74.0 (72.0, 77.0) | 74.0 (72.0, 77.0) | 74.0 (72.0, 77.0) | 75.0 (72.0, 77.0) | 74.0 (72.0, 77.0) | 75.2 (72.0, 79.2) | 76.05 (73.1, 79.6) | 75.10 (71.7, 79.0) | 75.5 (72.1, 79.2) | 74.8 (71.7, 79.0) | 60.6 (58.0, 63.2) | 61.3 (58.0, 63.4) | 60.8 (58.3, 63.6) | 60.83 (58.5, 63.1) | 60.2 (57.9, 62.9) | 85.5 (85.3, 85.9) | 85.6 (85.3, 85.9) | 85.6 (85.3, 85.9) | 85.5 (85.3, 85.8) | 85.5 (85.1, 85.8) |
| Women, % (*n*) | 50.9 (1297) | 49.6 (495) | 51.0 (294) | 50.2 (223) | 53.8 (285) | 52.2 (898) | 57.0 (146) | 50.1 (216) | 52.8 (237) | 51.2 (299) | 52.4 (315) | 55.8 (43) | 55.3 (57) | 55.6 (89) | 48.3 (126) | 59.9 (429) | 67.5 (135) | 65.5 (127) | 62.0 (88) | 43.9 (79) |
| Education, % (*n*) | | |  |  |  |  |  |  |  |  |  |  |  |  |  |  |  |  |  |  |
| Lower | 22.4 (569) | 22.7 (225) | 20.5 (118) | 21.3 (94) | 24.9 (132) | 35.3 (607) | 41.0 (105) | 35.0 (151) | 33.9 (152) | 34.1 (199) | 24.0 (144) | 16.9 (13) | 27.2 (28) | 19.4 (31) | 27.6 (72) | 63.7 (456) | 65.5 (131) | 71.1 (138) | 56.3 (80) | 59.4 (107) |
| Medium | 32.4 (824) | 34.1 (339) | 34.9 (201) | 28.3 (125) | 30.0 (159) | 39.4 (678) | 42.6 (109) | 38.5 (166) | 38.3 (172) | 39.6 (231) | 63.1 (379) | 66.2 (51) | 57.3 (59) | 66.9 (107) | 62.1 (162) | 31.6 (226) | 32.5 (65) | 24.7 (48) | 35.2 (50) | 35.0 (63) |
| Higher | 45.2 (1148) | 43.2 (429) | 44.6 (257) | 50.5 (223) | 45.1 (239) | 25.3 (435) | 16.4 (42) | 26.5 (114) | 27.8 (125) | 26.4 (154) | 13.0 (78) | 16.9 (13) | 15.5 (16) | 13.8 (22) | 10.3 (27) | 4.7 (34) | 2.0 (4) | 4.1 (8) | 8.5 (12) | 5.6 (10) |
| *Anthropometry* | | |  |  |  |  |  |  |  |  |  |  |  |  |  |  |  |  |  |  |
| Height, m, mean (SD) | 1.66 (0.09) | 1.68 (0.10) | 1.66 (0.09) | 1.66 (0.09) | 1.65 (0.09) | 1.61 (0.09) | 1.61 (0.09) | 1.62 (0.09) | 1.62 (0.09) | 1.61 (0.09) | 1.73 (0.09) | 1.73 (0.09) | 1.73 (0.09) | 1.73 (0.09) | 1.72 (0.09) | 1.62 (0.08) | 1.61 (0.08) | 1.61 (0.07) | 1.61 (0.07) | 1.64 (0.07) |
| BMI, kg/m^2^, mean (SD) | 27.2 (4.8) | 27.8 (4.4) | 27.2 (4.8) | 26.8 (5.0) | 26.5 (5.3) | 27.8 (4.5) | 28.8 (4.3) | 28.4 (4.0) | 27.9 (4.4) | 26.9 (4.8) | 27.0 (4.6) | 28.1 (4.3) | 27.3 (4.4) | 26.8 (4.6) | 26.6 (5.0) | 24.5 (4.4) | 25.9 (4.7) | 24.4 (4.7) | 24.0 (3.6) | 23.5 (3.8) |
| *Lifestyle* | | |  |  |  |  |  |  |  |  |  |  |  |  |  |  |  |  |  |  |
| Smokers, % (*n*) | 9.3 (236) | 8.2 (82) | 8.5 (49) | 9.5 (42) | 11.9 (63) | 6.8 (117) | 7.4 (19) | 8.4 (36) | 6.9 (31) | 5.3 (31) | 14.0 (84) | 14.3 (11) | 15.5 (16) | 14.5 (23) | 13.1 (34) | 6.0 (43) | 5.5 (11) | 4.6 (9) | 5.6 (8) | 8.3 (15) |
| Alcohol drinkers, % (*n*) | 36.4 (927) | 34.4 (343) | 35.2 (203) | 40.8 (181) | 37.7 (200) | 48.0 (825) | 41.0 (105) | 46.6 (201) | 49.4 (222) | 50.9 (297) | 79.2 (476) | 76.6 (59) | 80.6 (83) | 85.0 (136) | 75.9 (198) | 36.9 (264) | 37.0 (74) | 36.6 (71) | 28.2 (40) | 43.9 (79) |
| Physical Activity, % (*n*) | | |  |  |  |  |  |  |  |  |  |  |  |  |  |  |  |  |  |  |
| Lower | 33.2 (846) | 36.8 (367) | 29.2 (168) | 34.9 (155) | 29.5 (156) | 33.3 (572) | 37.9 (97) | 33.0 (142) | 33.6 (151) | 31.2 (182) | 33.0 (198) | 45.5 (35) | 30.1 (31) | 32.1 (51) | 31.0 (81) | 25.3 (181) | 29.5 (59) | 26.8 (52) | 22.5 (32) | 21.1 (38) |
| Medium | 33.1 (843) | 32.4 (323) | 37.2 (214) | 30.6 (136) | 32.1 (170) | 33.3 (573) | 36.3 (93) | 33.3 (143) | 34.3 (154) | 31.3 (183) | 33.3 (200) | 39.0 (30) | 31.1 (32) | 37.1 (59) | 30.3 (79) | 37.2 (266) | 38.5 (77) | 35.6 (69) | 43.7 (62) | 32.2 (58) |
| Higher | 33.6 (856) | 30.8 (307) | 33.6 (193) | 34.5 (153) | 38.4 (203) | 33.4 (574) | 25.8 (66) | 33.7 (145) | 32.1 (144) | 37.5 (219) | 33.7 (202) | 15.6 (12) | 38.8 (40) | 30.8 (49) | 38.7 (101) | 37.6 (269) | 32.0 (64) | 37.6 (73) | 33.8 (48) | 46.7 (84) |
| *Health* | | |  |  |  |  |  |  |  |  |  |  |  |  |  |  |  |  |  |  |
| Multimorbidity, % (*n*) | 42.6 (1084) | 41.0 (409) | 45.3 (261) | 44.1 (196) | 41.1 (218) | 49.7 (723) | 56.7 (122) | 50.1 (179) | 49.7 (186) | 46.4 (236) | 64.6 (388) | 71.4 (55) | 67.0 (69) | 68.1 (109) | 59.4 (155) | 70.8 (507) | 75.0 (150) | 68.6 (133) | 71.8 (102) | 67.8 (122) |
| Cognition, % (*n*) | | |  |  |  |  |  |  |  |  |  |  |  |  |  |  |  |  |  |  |
| Lower | 32.0 (764) | 31.8 (295) | 29.3 (159) | 33.7 (142) | 33.7 (168) | 28.1 (481) | 29.0 (74) | 29.2 (126) | 28.2 (126) | 26.7 (155) | 23.1 (139) | 23.4 (18) | 29.1 (30) | 24.4 (39) | 19.9 (52) | 31.1 (223) | 33.0 (66) | 32.5 (63) | 31.0 (44) | 27.8 (50) |
| Medium | 36.6 (876) | 37.9 (352) | 36.8 (200) | 34.4 (145) | 35.9 (179) | 51.1 (875) | 51.8 (132) | 47.8 (206) | 49.9 (223) | 54.0 (314) | 50.6 (304) | 50.6 (39) | 50.5 (52) | 43.1 (69) | 55.2 (144) | 29.6 (212) | 26.0 (52) | 32.0 (62) | 27.5 (39) | 32.8 (59) |
| Higher | 31.4 (751) | 30.4 (282) | 33.9 (184) | 31.8 (134) | 30.3 (151) | 20.9 (358) | 19.2 (49) | 23.0 (99) | 21.9 (98) | 19.3 (112) | 26.3 (158) | 26.0 (20) | 20.4 (21) | 32.5 (52) | 24.9 (65) | 39.2 (281) | 41.0 (82) | 35.6 (69) | 41.5 (59) | 39.4 (71) |
| *Dietary intake* | | |  |  |  |  |  |  |  |  |  |  |  |  |  |  |  |  |  |  |
| Energy intake, MJ, mean (SD) | 7.66 (2.75) | 5.58 (1.72) | 7.59 (1.83) | 8.79 (1.92) | 10.70 (2.36) | 7.70 (2.10) | 5.79 (1.46) | 6.98 (1.58) | 7.71 (1.67) | 9.07 (2.05) | 8.93 (2.48) | 5.83 (1.22) | 7.56 (1.61) | 8.52 (1.50) | 10.63 (2.19) | 7.05 (2.11) | 5.33 (1.48) | 6.89 (1.51) | 7.45 (1.58) | 8.82 (2.09) |
| Energy intake, z-score, mean (SD) | 0.00 (1.00) | -0.76 (0.63) | -0.02 (0.67) | 0.42 (0.70) | 1.11 (0.86) | 0.00 (1.00) | -0.91 (0.70) | -0.34 (0.75) | 0.00 (0.80) | 0.65 (0.98) | 0.01 (1.00) | -1.24 (0.49) | -0.55 (0.65) | -0.16 (0.60) | 0.69 (0.88) | 0.00 (1.00) | -0.82 (0.70) | -0.08 (0.71) | 0.19 (0.75) | 0.84 (0.99) |
| Protein, g/d, mean (SD) | 65.5 (26.0) | 43.2 (11.6) | 63.2 (8.9) | 76.4 (10.8) | 101.0 (22.7) | 74.2 (21.2) | 47.1 (8.4) | 62.4 (8.4) | 74.5 (10.3) | 94.5 (18.0) | 83.6 (24.0) | 49.4 (8.9) | 66.5 (9.4) | 79.5 (10.8) | 103.0 (18.5) | 64.2 (22.4) | 42.6 (9.0) | 57.3 (9.0) | 68.7 (8.4) | 92.3 (19.5) |
| Protein, % energy, mean (SD) | 14.5 (3.0) | 13.3 (2.7) | 14.5 (2.92) | 15.0 (2.8) | 16.1 (3.1) | 16.4 (3.4) | 14.1 (2.7) | 15.5 (3.2) | 16.7 (3.0) | 17.9 (3.4) | 15.8 (2.5) | 14.5 (2.5) | 15.1 (2.6) | 15.9 (2.3) | 16.5 (2.4) | 15.5 (3.5) | 14.0 (3.4) | 14.3 (2.8) | 15.9 (2.8) | 18.0 (3.5) |
| Protein, g/kg aBW/d, mean (SD) | 0.94 (0.38) | 0.60 (0.14) | 0.90 (0.06) | 1.09 (0.06) | 1.49 (0.29) | 1.11 (0.31) | 0.69 (0.09) | 0.90 (0.06) | 1.10 (0.06) | 1.44 (0.22) | 1.17 (0.32) | 0.67 (0.10) | 0.91 (0.05) | 1.10 (0.06) | 1.45 (0.21) | 1.01 (0.33) | 0.65 (0.11) | 0.91 (0.06) | 1.10 (0.06) | 1.44 (0.27) |
| *Muscle strength* | | |  |  |  |  |  |  |  |  |  |  |  |  |  |  |  |  |  |  |
| Grip strength, kg, median (IQR) | 28.0 (22.0, 37.0) | 29.0 (22.0, 38.0) | 28.0 (22.0, 37.0) | 29.0 (22.0, 36.0) | 28.0 (21.0, 36.0) | 29.0 (23.9, 35.2) | 27.8 (23.4, 34.2) | 29.4 (23.9, 35.4) | 28.8 (23.9, 35.0) | 29.4 (24.8, 35.7) | 31.5 (24.5, 43.5) | 31.0 (23.5, 42.5) | 30.0 (23.3, 43.0) | 29.8 (24.0, 39.8) | 33.5 (25.5, 45.5) | 17.9 (13.4, 23.9) | 16.6 (13.5, 21.8) | 16.4 (11.5, 22.3) | 18.0 (13.8, 23.8) | 20.4 (15.4, 26.2) |
| Grip strength, z-score, mean (SD) | 0.00 (1.00) | 0.06 (1.00) | -0.05 (0.98) | -0.02 (1.02) | -0.04 (1.00) | 0.00 (1.00) | -0.10 (0.92) | 0.02 (1.00) | -0.03 (0.96) | 0.05 (1.06) | 0.00 (1.00) | -0.09 (1.04) | -0.13 (1.12) | -0.05 (0.97) | 0.11 (0.95) | 0.00 (1.00) | 0.00 (0.97) | -0.12 (1.03) | 0.05 (1.01) | 0.09 (0.98) |

Cognition was assessed with the Mini-Mental State Examination. Smokers and alcohol drinkers represent current consumers. Z-scores and tertiles are cohort-specific and z-scores for grip strength are also sex-specific. aBW, adjusted body weight; BMI, body mass index; Health ABC, Health, Aging and Body Composition Study; IQR, interquartile range; LASA, Longitudinal Aging Study Amsterdam; MMSE, Mini-Mental State Examination; N85+, Newcastle 85+ Study; NuAge, Quebec Longitudinal Study on Nutrition and Successful Aging; SD, standard deviation; y, years.

**Supplementary Table 4.** Health and sociodemographic characteristics of participants by wave of follow-up^1^.

|  | **Baseline** (*n*=5584) | **Wave 2** (*n*=4867) | **Wave 3** (*n*=3880) | **Wave 4** (*n*=3330) | **Wave 5** (*n*=1508) |
| --- | --- | --- | --- | --- | --- |
| *Sociodemographic* |  |  |  |  |  |
| Age, y, median (IQR) | 75.0 (71.6, 79.0) | 76.0 (73.0, 80.1) | 78.1 (75.9, 82.0) | 80.0 (77.0, 83.1) | 82.0 (80.0, 84.0) |
| Women, % (*n*) | 52.6 (2939) | 52.9 (2576) | 53.7 (2085) | 53.5 (1783) | 54.4 (821) |
| Cohort, % (*n*) |  |  |  |  |  |
| Health ABC | 45.6 (2547) | 46.2 (2250) | 51.1 (1982) | 50.5 (1681) | 100.0 (1508) |
| NuAge | 30.8 (1720) | 30.9 (1505) | 37.7 (1464) | 40.8 (1358) | 0.0 (0) |
| LASA | 10.8 (601) | 11.1 (542) | 0.0 (0) | 0.0 (0) | 0.0 (0) |
| N85+ | 12.8 (716) | 11.7 (570) | 11.2 (434) | 8.7 (291) | 0.0 (0) |
| Education, % (*n*) |  |  |  |  |  |
| Lower | 31.8 (1776) | 31.1 (1510) | 31.1 (1205) | 29.8 (992) | 18.8 (283) |
| Medium | 37.8 (2107) | 37.9 (1843) | 34.3 (1331) | 34.7 (1156) | 31.9 (480) |
| Higher | 30.4 (1695) | 31.0 (1508) | 34.6 (1341) | 35.4 (1179) | 49.3 (743) |
| *Anthropometry* |  |  |  |  |  |
| Height, m, mean (SD) | 1.65 (0.10) | 1.65 (0.10) | 1.64 (0.09) | 1.64 (0.09) | 1.66 (0.09) |
| BMI, kg/m^2^, mean (SD) | 27.0 (4.7) | 27.1 (4.7) | 27.1 (4.8) | 27.1 (4.8) | 27.1 (4.9) |
| *Lifestyle* |  |  |  |  |  |
| Smokers, % (*n*) | 8.6 (480) | 7.4 (359) | 6.0 (232) | 5.7 (185) | 6.6 (99) |
| Alcohol drinkers, % (*n*) | 44.6 (2492) | 45.8 (2218) | 41.7 (1603) | 43.4 (1425) | 38.9 (586) |
| Physical Activity, % (*n*) |  |  |  |  |  |
| Lower | 32.2 (1797) | 36.0 (1744) | 41.9 (1592) | 41.3 (1304) | 49.1 (727) |
| Medium | 33.7 (1882) | 40.7 (1971) | 32.6 (1240) | 33.1 (1047) | 30.6 (453) |
| Higher | 34.1 (1901) | 23.2 (1125) | 25.5 (971) | 25.6 (810) | 20.4 (302) |
| *Health* |  |  |  |  |  |
| Multimorbidity, % (*n*) | 50.8 (2702) | 55.3 (2668) | 55.5 (2131) | 56.9 (1847) | 57.8 (872) |
| Cognition, % (*n*) |  |  |  |  |  |
| Lower | 29.6 (1607) | 30.8 (1382) | 29.8 (1109) | 36.1 (1159) | 36.9 (532) |
| Medium | 41.8 (2267) | 39.1 (1754) | 38.3 (1428) | 39.7 (1275) | 32.0 (462) |
| Higher | 28.6 (1548) | 30.1 (1350) | 31.9 (1187) | 24.2 (776) | 31.1 (449) |
| *Dietary intake* |  |  |  |  |  |
| Energy intake, z-score, mean (SD) | 0.00 (1.00) | 0.03 (1.01) | 0.01 (1.00) | 0.02 (1.02) | -0.03 (0.98) |
| Protein intake, g/kg BW/d, % (*n*) |  |  |  |  |  |
| <0.8 | 33.4 (1867) | 33.6 (1624) | 35.9 (1379) | 35.7 (1171) | 46.2 (696) |
| 0.8-<1.0 | 23.3 (1300) | 23.4 (1130) | 23.2 (891) | 22.6 (740) | 21.1 (318) |
| 1.0-<1.2 | 19.4 (1084) | 19.0 (920) | 18.5 (712) | 18.7 (615) | 14.7 (221) |
| ≥1.2 | 23.9 (1333) | 24.0 (1159) | 22.5 (864) | 23.0 (755) | 18.1 (273) |
| Protein intake, g/kg aBW/d, % (*n*) |  |  |  |  |  |
| <0.8 | 27.4 (1530) | 27.8 (1320) | 29.6 (1135) | 29.0 (946) | 40.1 (605) |
| 0.8-<1.0 | 23.4 (1304) | 23.2 (1103) | 24.5 (939) | 23.5 (765) | 22.1 (334) |
| 1.0-<1.2 | 21.4 (1195) | 20.9 (990) | 20.2 (774) | 21.3 (695) | 16.6 (250) |
| ≥1.2 | 27.8 (1555) | 28.1 (1334) | 25.8 (992) | 26.2 (855) | 21.2 (319) |
| *Muscle strength* |  |  |  |  |  |
| Grip strength, kg, median (IQR) | 28.0 (21.5, 35.8) | 27.1 (21.0, 35.0) | 26.0 (20.0, 33.4) | 25.3 (19.8, 32.2) | 25.0 (20.0, 32.0) |
| Grip strength, z-score, mean (SD) | 0.00 (1.00) | -0.10 (0.99) | -0.21 (1.01) | -0.36 (1.01) | -0.45 (0.94) |

^1^ Not all waves are at the same time of follow-up among cohorts. In Health ABC the necessary variables were available at year 2 (operationalized as baseline), 4 (wave 2), 6 (wave 3), 8 (wave 4) and 10 (wave 5). In NuAge data were available at year 1 (baseline), 2 (wave 2), 3 (wave 3) and 4 (wave 4). In LASA variables were available at wave 3B (baseline) and at wave I after 3 years (wave 2). The Newcastle 85+ has data at baseline, after 18 months (wave 2), after 36 months (wave 3) and after 60 months (wave 4). Cognition was assessed with the Mini-Mental State Examination. Smokers and alcohol drinkers represent current consumers. Z-scores and tertiles are cohort-specific and z-scores for grip strength are also sex-specific. aBW, adjusted body weight; Health ABC, Health, Aging and Body Composition Study; IQR, interquartile range; LASA, Longitudinal Aging Study Amsterdam; MMSE, Mini-Mental State Examination; N85+, Newcastle 85+ Study; NuAge, Quebec Longitudinal Study on Nutrition and Successful Aging; Prot, protein; SD, standard deviation; y, years.

**Supplementary Table 5.** Health and sociodemographic characteristics of participants by protein intake and physical activity at baseline.

| **Prot (g/kg aBW/d)** | **<0.8** (*n*=1530) | | | **0.8-<1.0** (*n*=1302) | | | **1.0-<1.2** (*n*=1194) | | | **≥1.2** (*n*=1554) | | |
| --- | --- | --- | --- | --- | --- | --- | --- | --- | --- | --- | --- | --- |
| **Physical Activity** | **Lower** (*n*=558) | **Medium** (*n*=523) | **Higher** (*n*=449) | **Lower** (*n*=393) | **Medium** (*n*=458) | **Higher** (*n*=451) | **Lower** (*n*=389) | **Medium** (*n*=411) | **Higher** (*n*=394) | **Lower** (*n*=457) | **Medium** (*n*=490) | **Higher** (*n*=607) |
| *Sociodemographic* |  |  |  |  |  |  |  |  |  |  |  |  |
| Age, y, median (IQR) | 75.0 (72.0, 79.0) | 75.0 (72.0, 79.0) | 75.0 (72.0, 78.0) | 75.7 (72.5, 80.0) | 75.0 (72.0, 79.0) | 74.0 (71.2, 78.6) | 75.0 (71.0, 79.2) | 75.0 (71.0, 79.9) | 74.8 (71.0, 78.0) | 74.0 (70.8, 79.0) | 75.0 (71.0, 79.0) | 73.3 (70.5, 78.0) |
| Women, % (*n*) | 60.9 (353) | 56.4 (304) | 41.7 (192) | 60.0 (240) | 56.2 (266) | 45.7 (210) | 57.7 (229) | 57.8 (244) | 45.0 (179) | 53.8 (254) | 55.7 (279) | 45.9 (284) |
| Cohort, % (*n*) |  |  |  |  |  |  |  |  |  |  |  |  |
| Health ABC | 67.1 (389) | 62.7 (338) | 68.9 (317) | 42.8 (171) | 47.8 (226) | 43.5 (200) | 40.1 (159) | 33.9 (143) | 39.2 (156) | 35.4 (167) | 35.7 (179) | 34.4 (213) |
| NuAge | 16.7 (97) | 17.3 (93) | 14.3 (66) | 36.0 (144) | 30.4 (144) | 31.5 (145) | 38.3 (152) | 36.5 (154) | 36.2 (144) | 38.6 (182) | 36.7 (184) | 35.5 (220) |
| LASA | 6.0 (35) | 5.8 (31) | 2.8 (13) | 8.0 (32) | 7.2 (34) | 9.1 (42) | 13.6 (54) | 14.7 (62) | 12.6 (50) | 18.0 (85) | 15.8 (79) | 16.5 (102) |
| N85+ | 10.2 (59) | 14.3 (77) | 13.9 (64) | 13.2 (53) | 14.6 (69) | 15.9 (73) | 8.1 (32) | 14.9 (63) | 12.1 (48) | 8.1 (38) | 11.8 (59) | 13.6 (84) |
| Education, % (*n*) |  |  |  |  |  |  |  |  |  |  |  |  |
| Lower | 30.6 (177) | 30.7 (165) | 32.2 (148) | 33.5 (134) | 30.7 (145) | 35.4 (163) | 29.5 (117) | 30.3 (128) | 30.2 (120) | 32.4 (153) | 32.1 (161) | 33.8 (209) |
| Medium | 40.8 (236) | 36.7 (197) | 32.0 (147) | 38.2 (153) | 35.9 (170) | 35.4 (163) | 36.6 (145) | 38.6 (163) | 38.3 (152) | 41.1 (194) | 40.9 (205) | 37.2 (230) |
| Higher | 28.5 (165) | 32.6 (175) | 35.9 (165) | 28.2 (113) | 33.4 (158) | 29.1 (134) | 33.8 (134) | 31.0 (131) | 31.5 (125) | 26.5 (125) | 26.9 (135) | 29.1 (180) |
| *Anthropometry* |  |  |  |  |  |  |  |  |  |  |  |  |
| Height, m, mean (SD) | 1.65 (0.10) | 1.66 (0.10) | 1.68 (0.10) | 1.63 (0.10) | 1.65 (0.09) | 1.66 (0.09) | 1.64 (0.10) | 1.64 (0.10) | 1.66 (0.09) | 1.64 (0.11) | 1.64 (0.10) | 1.65 (0.09) |
| BMI, kg/m^2^, mean (SD) | 28.2 (4.8) | 27.8 (4.5) | 26.9 (3.9) | 28.2 (5.2) | 26.7 (4.5) | 26.8 (4.3) | 27.6 (5.2) | 26.8 (4.5) | 26.3 (4.2) | 27.4 (5.6) | 26.1 (4.8) | 25.7 (4.5) |
| *Lifestyle* |  |  |  |  |  |  |  |  |  |  |  |  |
| Smokers, % (*n*) | 12.0 (69) | 7.4 (40) | 3.9 (18) | 11.2 (45) | 6.6 (31) | 7.8 (36) | 12.9 (51) | 6.4 (27) | 6.8 (27) | 10.0 (47) | 9.0 (45) | 8.4 (52) |
| Alcohol drinkers, % (*n*) | 32.8 (190) | 38.6 (208) | 43.0 (198) | 36.0 (144) | 46.1 (218) | 44.6 (205) | 46.3 (184) | 47.9 (202) | 51.0 (203) | 46.6 (220) | 52.5 (263) | 49.8 (308) |
| *Health* |  |  |  |  |  |  |  |  |  |  |  |  |
| Multimorbidity, % (*n*) | 54.3 (306) | 48.7 (256) | 44.9 (201) | 60.2 (227) | 51.5 (229) | 47.1 (206) | 61.0 (227) | 53.9 (212) | 43.9 (165) | 54.6 (248) | 49.6 (237) | 45.6 (267) |
| Cognition, % (*n*) |  |  |  |  |  |  |  |  |  |  |  |  |
| Lower | 35.3 (195) | 31.0 (161) | 26.5 (116) | 29.9 (116) | 30.7 (141) | 29.1 (131) | 36.4 (143) | 26.4 (109) | 27.3 (105) | 33.8 (155) | 27.4 (133) | 25.3 (155) |
| Medium | 41.4 (229) | 38.2 (198) | 38.2 (167) | 45.4 (176) | 37.4 (172) | 40.7 (183) | 41.7 (164) | 40.7 (168) | 39.5 (152) | 44.1 (202) | 45.6 (221) | 46.5 (285) |
| Higher | 23.3 (129) | 30.8 (160) | 35.2 (154) | 24.7 (96) | 32.0 (147) | 30.2 (136) | 21.9 (86) | 32.9 (136) | 33.2 (128) | 22.1 (101) | 27.0 (131) | 28.2 (173) |
| *Dietary intake* |  |  |  |  |  |  |  |  |  |  |  |  |
| Energy intake, z-score, mean (SD) | -0.9 (0.6) | -0.8 (0.6) | -0.7 (0.7) | -0.2 (0.7) | -0.2 (0.7) | -0.1 (0.7) | 0.1 (0.8) | 0.1 (0.7) | 0.3 (0.8) | 0.7 (0.9) | 0.8 (0.9) | 1.0 (1.0) |
| Protein, g/kg aBW/d, mean (SD) | 0.6 (0.1) | 0.6 (0.1) | 0.6 (0.1) | 0.9 (0.1) | 0.9 (0.1) | 0.9 (0.1) | 1.1 (0.1) | 1.1 (0.1) | 1.1 (0.1) | 1.4 (0.2) | 1.5 (0.2) | 1.5 (0.3) |
| *Muscle strength* |  |  |  |  |  |  |  |  |  |  |  |  |
| Grip strength, kg, median (IQR) | 25.5 (20.0, 33.4) | 27.1 (21.0, 35.7) | 30.4 (23.0, 38.0) | 25.8 (19.1, 33.0) | 27.1 (20.7, 35.0) | 29.0 (22.0, 36.0) | 26.0 (20.5, 34.0) | 28.1 (21.3, 34.2) | 29.3 (23.5, 36.0) | 27.6 (20.7, 35.6) | 28.0 (22.8, 36.0) | 30.0 (23.9, 36.8) |
| Grip strength, z-score, mean (SD) | -0.15 (1.00) | 0.05 (1.02) | 0.18 (0.91) | -0.19 (1.04) | -0.05 (1.00) | 0.08 (0.97) | -0.21 (1.03) | 0.05 (0.95) | 0.10 (0.96) | -0.15 (1.09) | 0.04 (0.96) | 0.17 (0.97) |

Cognition was assessed with the Mini-Mental State Examination. Smokers and alcohol drinkers represent current consumers. Z-scores and tertiles are cohort-specific and z-scores for grip strength are also sex-specific. aBW, adjusted body weight; BMI, body mass index; Health ABC, Health, Aging and Body Composition Study; IQR, interquartile range; LASA, Longitudinal Aging Study Amsterdam; N85+, Newcastle 85+ Study; NuAge, Quebec Longitudinal Study on Nutrition and Successful Aging; Prot, protein; SD, standard deviation; y, years.

**Supplementary Table 6.** Association between protein intake (g/kg non-adjusted body weight/d) or percentage of energy from protein (% total energy) at baseline and grip strength (sex and cohort-specific z-score) over time.

|  | **β Coefficient** | **95% Lower limit** | **95% Upper limit** |
| --- | --- | --- | --- |
| **Protein, g/kg BW/d** |  |  |  |
| Time, y | -0.015 | -0.024 | -0.004 |
| Protein intake, g/kg BW/d |  |  |  |
| <0.8 (ref.) | 0 | 0 | 0 |
| 0.8-<1.0 | -0.006 | -0.036 | 0.028 |
| 1.0-<1.2 | -0.016 | -0.064 | 0.018 |
| >1.2 | -0.030 | -0.069 | 0.015 |
| Time x Protein intake |  |  |  |
| Time x <0.8 (ref.) | 0 | 0 | 0 |
| Time x 0.8-<1.0 | -0.011 | -0.023 | -0.001 |
| Time x 1.0-<1.2 | -0.010 | -0.022 | 0.00 |
| Time x ≥1.2 | -0.004 | -0.015 | 0.003 |
| **Protein, % Energy** |  |  |  |
| Time, y | -0.004 | -0.028 | 0.013 |
| Protein, % Energy | -0.003 | -0.007 | 0.001 |
| Time x Protein, % Energy | -0.001 | -0.002 | 0 |
| **Protein, g/MJ** |  |  |  |
| Time, y | -0.004 | -0.021 | 0.011 |
| Protein, g/MJ | -0.004 | -0.013 | 0.002 |
| Time x Protein, g/MJ | -0.002 | -0.004 | 0 |

The model is the fully adjusted model 4 or panel Fig. 1-D but with a term for non-adjusted protein intake (g/kg bodyweight (BW)/d) or a term for percentage of total energy (%) from protein or a term for protein intake per MJ of energy intake (g/MJ) instead of protein intake (g) per adjusted BW/d. The models include terms for sex, age, education, height, smoking, and alcohol intake, cognition, multimorbidity and physical activity. The model for protein (g/kg BW/d) includes a term for energy intake while the model for protein (% of total energy) and protein per MJ does not include a term for energy intake and includes one for weight (n=18643 person-years). Results are presented as β coefficients and 95%CI.

**Supplementary Fig. 1.** Flowchart of the four longitudinal ageing cohorts included and the exclusion criteria for the analytic sample.

**
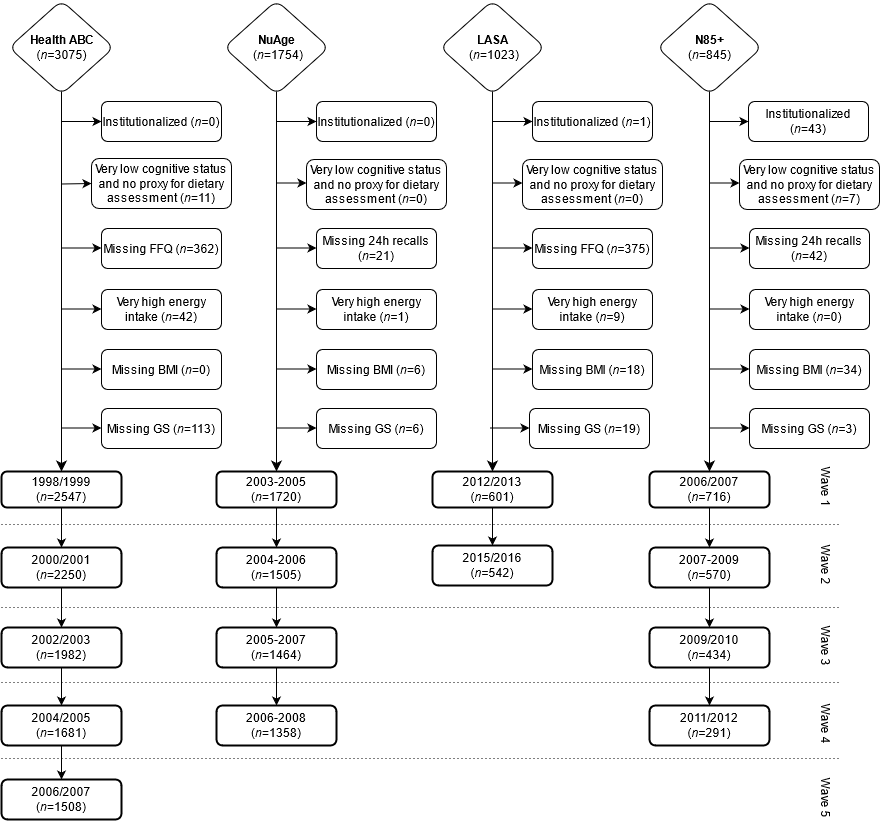
**

Very low cognitive status was defined by a Mini-Mental State Examination score <18 or having dementia. Very high energy intakes were defined as >3500 kcal/d for women or >4000 kcal/d for men. The term wave is not synonymous with homogeneity between cohorts and the different characteristics are described in Supplementary Table 3. BMI, body mass index; FFQ, food frequency questionnaire; GS, grip strength; Health ABC, Health, Aging and Body Composition Study; LASA, Longitudinal Aging Study Amsterdam; N85+, Newcastle 85+ Study; NuAge, Quebec Longitudinal Study on Nutrition and Successful Aging.

**Supplementary Fig. 2.** Cumulative hazards for all-cause mortality over time by protein intake (g/kg aBW/d).


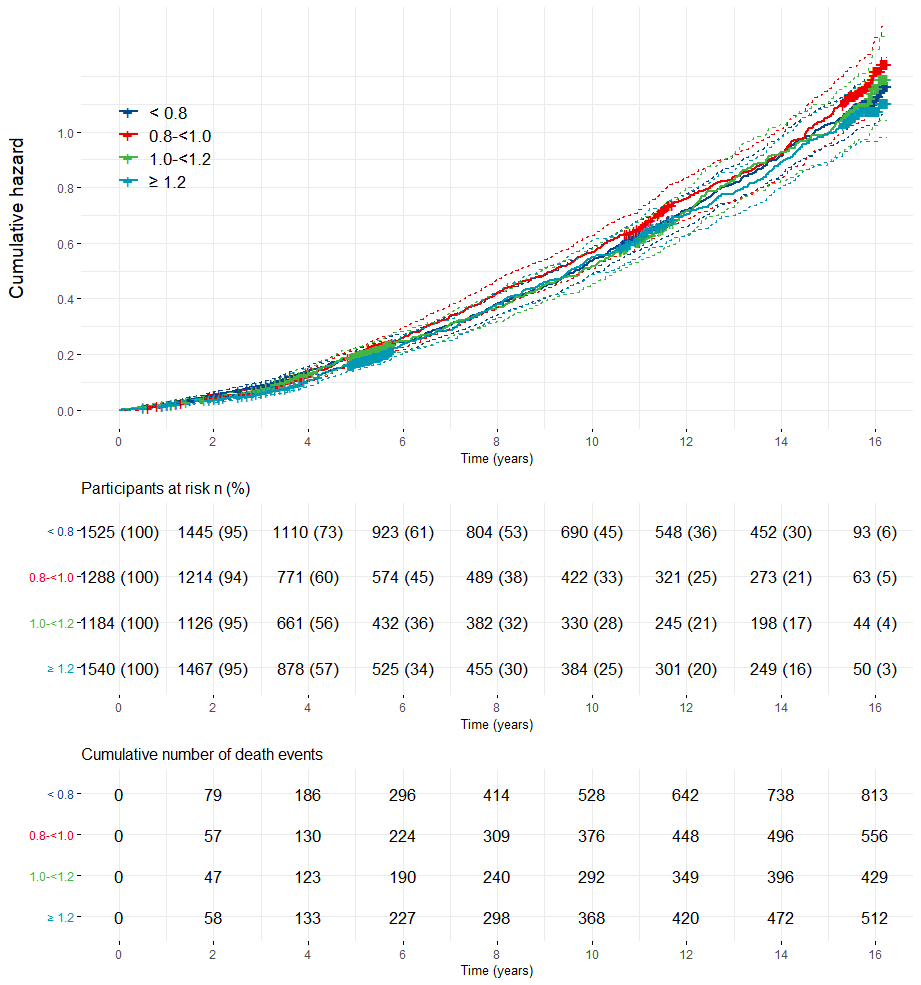


Percentage (%) participants at risk are participants that are still in the study and alive over time. g/kg aBW/d; grams of protein per kilogram of adjusted body weight per day.

**Supplementary Fig. 3.** Association between protein intake (g/kg aBW/d) at baseline and grip strength (sex and cohort specific z-score) over time excluding each of the cohorts used.


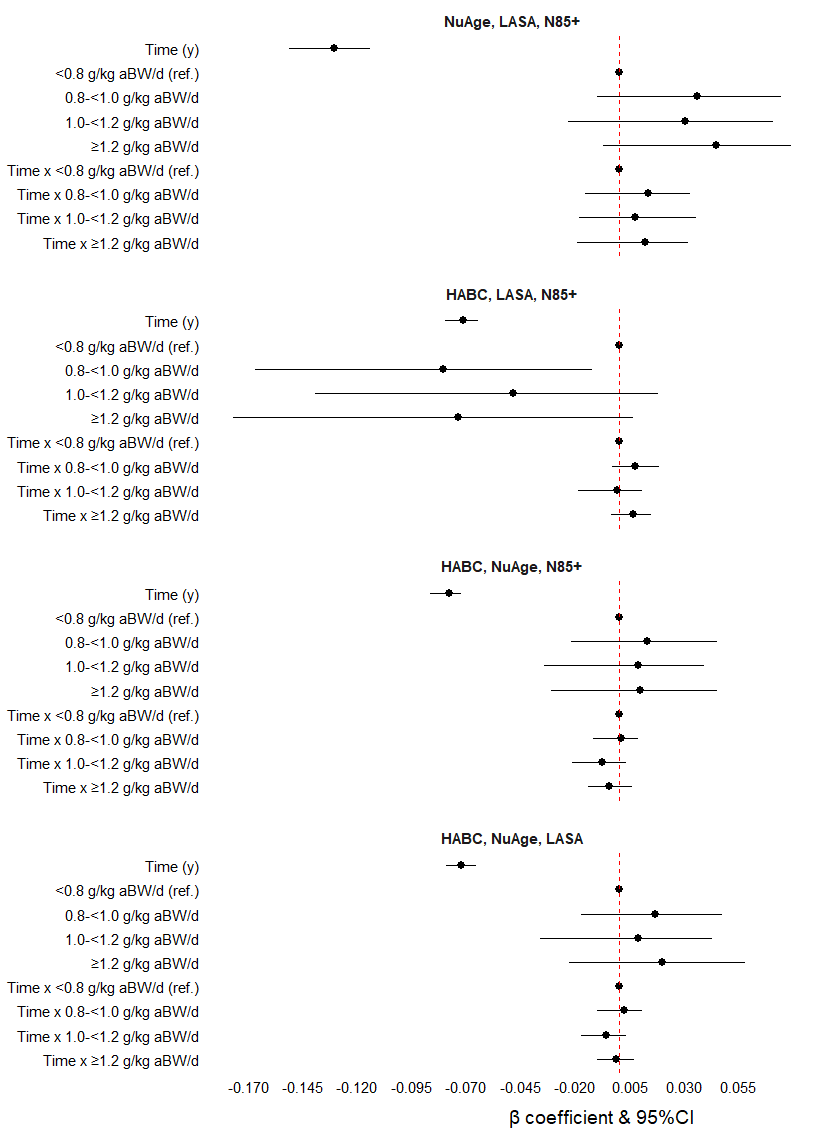


The models (adjusted for protein intake, sex, age, education, height, smoking, energy and alcohol intake, cognition, multimorbidity and physical activity) were re-run excluding one or another cohort (NuAge+LASA+N85+: *n*=8744 person-years; HABC+LASA+N85+: *n*=12985 person-years; HABC+NuAge+N85+: *n*=17500 person-years; HABC+ NuAge+LASA: *n*=16700 person-years). Results are presented as β coefficients and 95%CI in the x axis and the terms of interest in the y axis. CI, confidence interval; g/kg aBW/d, grams of protein per kilogram of adjusted body weight per day; Health ABC, Health, Aging and Body Composition Study; IQR, interquartile range; LASA, Longitudinal Aging Study Amsterdam; NuAge, Quebec Longitudinal Study on Nutrition and Successful Aging; N85+, Newcastle 85+ Study; ref, referent.

**References**

1. Folstein MF, Folstein SE, McHugh PR (1975) "Mini-mental state". A practical method for grading the cognitive state of patients for the clinician. J Psychiatr Res 12 (3):189-198. doi:10.1016/0022-3956(75)90026-6

2. Teng EL, Chui HC (1987) The Modified Mini-Mental State (3MS) examination. J Clin Psychiatry 48 (8):314-318
